# Efficacy and safety of remimazolam besylate versus propofol during hysteroscopy: single-centre randomized controlled trial

**DOI:** 10.1186/s12871-021-01373-y

**Published:** 2021-05-20

**Authors:** Xiaoqiang Zhang, Shuang Li, Jing Liu

**Affiliations:** Department of Anaesthesiology, Mengcheng County No. 1 People’s Hospital, Mengcheng, 233500 Anhui Province P. R. China

**Keywords:** Anaesthesia, Remimazolam besylate, Benzodiazepine, Propofol, Remifentanil, Hysteroscopy

## Abstract

**Background:**

Remimazolam besylate is a newer benzodiazepine with characteristics of quick onset of effects, short maintenance and recovery times without accumulation in tissues. This trial was conducted to confirm the efficacy and safety of remimazolam besylate versus propofol during hysteroscopy.

**Methods:**

Patients undergoing hysteroscopy were randomly assigned to either the remimazolam (Group R) or the propofol group (Group P). Group R was administered an induction dose of 0.2 mg/kg and a maintenance dosage of 1.0 mg/kg/h. In Group P, propofol was started at 1.5–2.0 mg/kg and then maintained at 3.0–6.0 mg/kg/h. After remimazolam besylate or propofol induction, remifentanil was infused using a target-controlled infusion system with a target concentration of 1.5 ng/ml and titrated during the procedure. The incidence rates of injection pain, low oxygen saturation (SpO_2_) and adverse effects in both groups were compared.

**Results:**

Eighty-two patients were included in this study. The incidence of adverse events in Group R (3.7%) was significantly lower than that in Group P (36.6%) (*p* < 0.001). The incidence of injection pain in Group P (80.5%) was much higher than that in Group R (2.4%) (*p* < 0.001). The incidence of other adverse events, such as low SpO_2_, bradycardia, and hypotension in Group R was lower than that in Group P (*p* < 0.05).

**Conclusions:**

Remimazolam besylate proves to be a safer alternative for anesthesia during hysteroscopy. Moreover, adverse events caused by propofol, such as low SpO_2_ and injection pain, are largely avoided.

**Trial registration:**

This study was approved by the Clinical Research Ethics Committee of Mengcheng County No. 1 People’s Hospital (2020MYL20003) and registered at http://www.chictr.org.cn (15/09/2020, ChiCTR-2000038252). The study protocol followed the CONSORT guidelines. The study protocol was performed in the relevant guidelines.

## Background

Hysteroscopy is one of the most common outpatient procedures in the diagnosis and treatment of endometrial and other intrauterine diseases. Most patients require anaesthetic intervention because they cannot tolerate the intense pain of cervical dilatation and endometrial curettage [1–3].

The commonly used anesthetic methods used for hysteroscopic surgery include propofol combined with opioids, propofol combined with dexmedetomidine, paracervical block, and local anaesthesia [4–6]. Among these, propofol combined with opioids is still the most commonly used method to control pain during hysteroscopy [7]. However, propofol has a high incidence of adverse events, such as injection pain, postoperative dizziness, and low pulse oximetry (SpO_2_).

Remimazolam besylate is a newer benzodiazepine with characteristics of quick onset of effects, short maintenance and recovery times. It does not accumulate in tissues; metabolism is independent of liver and kidney function without any major side effect. Furthermore, opioids can have a sedative effect in some endoscopic examinations [8–10].

This trial was conducted to confirm the efficacy and safety of remimazolam besylate versus propofol during hysteroscopy.

## Methods

### Ethics and registration

This study was approved by the Clinical Research Ethics Committee of Mengcheng County No. 1 People’s Hospital (2020MYL20003) and registered at http://www.chictr.org.cn (15/09/2020, ChiCTR-2000038252). The study protocol followed the CONSORT guidelines. The study protocol was performed in the relevant guidelines. Written informed consent was obtained from patients undergoing elective hysteroscopy at Mengcheng County No. 1 People’s Hospital from 15/09/2020 to 20/12/2020.

### Patient inclusion and exclusion criteria

The inclusion criteria of the patients were age between 18 and 65 years old, American Society of Anesthesiologists (ASA) physical status I or II and body mass index (BMI) of 19 to 30 kg/m^2^. Patients with history of alcoholism or allergy to general anaesthetic drugs, renal or liver diseases, communication difficulties, lactation or recent respiratory infections were excluded.

### Randomization

Patients were randomly assigned into the remimazolam group (Group R) and the propofol group (Group P) by computer generated randomization.

### Technique

All patients fasted routinely before surgery. On arrival in the operating room, the Bene View N15 monitor (Mindray Biomedical Electronics Co., Shenzhen, China) was connected to monitor the electrocardiogram (ECG), noninvasive blood pressure (NIBP) including systolic blood pressure (SBP) and diastolic blood pressure (DBP), SpO_2_, and heart rate (HR). All patients inhaled oxygen (2 L/min) through a Venturi oxygen mask, and all patients received an intravenous (IV) COX-2 inhibiter, flurbiprofen axetil 50 mg (Wuhan Docan Pharmaceutical Co., Ltd., China) for analgesic preconditioning before the start of hysteroscopy.

### Grouping and intervention

All patients in Group R were started at an induction dose of 0.2 mg/kg remimazolam besylate (Yichang Humanwell Pharmaceutical Co., Ltd., China) and a maintenance dosage of 1 mg/kg/h by continuous IV infusion until the loss of consciousness (LoC) [11]. The dose was based on a randomized phase IIb/III trial conducted by Japanese researchers in 2019 showed that the induction dose of remimazolam (0.2 mg/kg) was no less effective than propofol (2.0–2.5 mg/kg) when used as a general anaesthesia sedative [12]. When the Modified Observer’s Alertness/Sedation (MOAA/S) score was ≤2 [13], hysteroscopy was started. According to the MOAA/S score, supplemental remimazolam was added at 2.5 mg/dose, with no more than 5 doses administered within 15 min, according to the drug instructions of the supplemental drug program [14].

All patients in Group P were started at 1.5–2.0 mg/kg propofol (Fresenius Kabi AG, Austria). When the MOAA/S score was ≤2, hysteroscopy was started. Then, the infusion rate of propofol was maintained with a dosage of 3.0 mg/kg/h. According to the MOAA/S score, the injection speed of propofol was adjusted to 3.0–6.0 mg/kg/h [15].

After remimazolam besylate or propofol induction, infusion of remifentanil (Yichang Humanwell Pharmaceutical Co., Ltd.) in both the groups was started with TCI pump (Guangxi VERYARK Technology Co., Ltd., China), and the effective effect-site concentration (Ce) (Minto pharmacokinetic model) was 1.5 ng/ml [16]. Remifentanil was increased by 0.5 ng/ml when analgesia was insufficient (facial grimace, movement, SBP > 140 mmHg, heart rate (HR) > 100 beats/min (bpm) or sudden increase of more than 30 bpm over baseline) and was decreased by 0.5 ng/ml with signs of excessive analgesia (respiratory depression, hypotension, or bradycardia) [17].

### Outcomes

#### Primary outcome

The primary outcome of this study was the incidence of various adverse events, such as injection pain, low SpO_2_, bradycardia and hypotension (definitions in Table 1). These events were treated by injecting ephedrine or atropine intravenously, or mask ventilating.

**Table 1:** The definition and incidence of adverse events

Adverse events	Definitions	Group R (*N* = 41)	Group P (*n* = 41)	*p* value
Injection pain	‘Subjective’ assessment, patients verbally reported their pain by themselves after the first injection	1 (2.4%)	33 (80.5%)	< 0.001
Low SpO_2_	Intraoperative SpO_2_ ≤ 95%	4 (9.8%)	21 (51.2%)	< 0.001
Bradycardia	Intraoperative HR < 55 bpm	0 (0.0%)	1 (2.4%)	0.314
Hypotension	Intraoperative SBP<90 mmHg	1 (2.4%)	5 (12.2%)	0.090
Total incidence of adverse events		6 (3.7%)	60 (36.6%)	< 0.001
Postoperative dizziness	Appeared while patients stayed in the postanaesthesia care unit (PACU)	0 (0.0%)	10 (24.4%)	< 0.001
Body movement	Visible hand bending or head movement	15 (36.6%)	20 (48.8%)	0.264

Note: Values are presented as n (%); HR-heart rate; SBP-systolic blood pressure

#### Secondary outcomes

The incidence of body movement and postoperative dizziness (definitions in Table 2) were recorded. Patient data fluctuations included the mean arterial pressure (MAP) (MAP = (SBP + 2 × DBP) / 3), HR, SpO_2_, and MOAA/S score at pre-anaesthesia (T0), 2 min post induction (T1), cervical dilatation (T2), the end of the operation (T3), and awakening (T4). The duration of awakening and postanaesthesia care unit (PACU) length of stay were recorded.

**Table 2:** Demographic characteristics and clinical data for each group

	Group R (*n* = 41)	Group P (n = 41)
Age (years)	43.8 ± 8.0	45.2 ± 7.0
Height (cm)	159.6 ± 5.2	160.0 ± 4.9
Weight (kg)	62.8 ± 7.6	61.6 ± 8.2
BMI (kg/m^2^)	24.7 ± 2.7	24.1 ± 2.8
ASA (I/II) (n)	28/13	34/7
Duration of operation (min)	13.2 ± 4.2	12.6 ± 4.7
Duration of awakening (s)	199.0 ± 79.9	59.7 ± 1.2
PACU length of stay (min)	5.44 ± 1.0	6.3 ± 1.9
Total remimazolam (mg/kg)	0.4 ± 0.1	–
Total propofol (mg/kg)	–	2.5 ± 0.6
Total remifentanil (μg)	75.7 ± 15.2	73.2 ± 22.2

Note: Data indicate the mean ± SD or n; *ASA* American Society of Anesthesiologists; *BMI* body mass index, *PACU* post anaesthesia care unit

We searched relevant literature regarding remimazolam and found that MOAA/S scores were used as the evaluation method of anaesthetic depth in most of the studies. The guiding significance of using the bispectral index (BIS) to study remimazolam was not clear; therefore, we had no confidence in using BIS and chose the MOAA/S score instead.

### Sample size and statistical analysis

In the pilot study on the combined use of propofol and remifentanil in hysteroscopy, the incidence of various intraoperative adverse events was 30%. This result of our small sample pre-experiment indicated a clinically significant reduction in the incidence of adverse events to 5% by the use of remimazolam. A sample size of 41 participants in each group was calculated, and the significance level was 0.05 (a = 0.05). Given a 10% attrition rate, the strength was 80% (b = 0.20) [18].

Statistical analysis was performed using SPSS Statistics 17.0.1 (SPSS Inc., Chicago, IL). Normality test in SPSS statistics software was used for data analysis to determine whether the data were in accordance with a normal distribution. Normally distributed continuous variables are presented as the mean ± standard deviation and were analysed using Student’s t test. The Mann-Whitney U test was used for non-normally distributed continuous variables. Hemodynamic parameters were compared by repeated measures ANOVA. Categorical variables are expressed as a frequency (percentage) and were analysed using the Pearson chi-square test. The Wilcoxon Signed-Rank test was used to compare continuous variables. A *p* value < 0.05 was considered to indicate statistical significance.

## Results

**Fig. 1:**
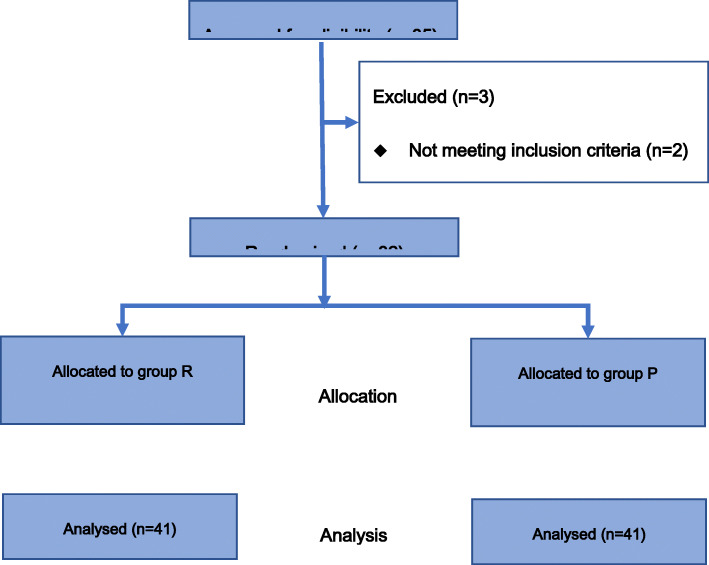
Flow diagram of the study

The study population comprised 82 randomly coded patients in Group R (*n* = 41) and Group P (n = 41) (Fig. 1).

### Demographic data and surgical characteristics

The demographic characteristics of the patients and surgical characteristics are given in Table 2. The characteristics were similar in both groups.

The operative durations were similar in the two groups (*p* > 0.05). The awakening time of Group R (199.0 ± 79.9 s) was significantly longer than that of Group P (59.7 ± 1.2 s) (*p* < 0.05). However, the PACU length of stay in Group R (5.44 ± 1.0 min) was significantly shorter than that in Group P (6.3 ± 1.9 min) (*p* < 0.05). The total remifentanil dose was not significantly different among the two groups (*p* > 0.05). The supplemental remimazolam dose was 10.8 ± 4.0 mg.

### Adverse events

Adverse events occurred on 6 (3.7%) occasions in group R and 60 (36.6%) occasions in group P (*p* < 0.001), with no serious adverse events or deaths occurring in the two groups (Table 2). The incidence of injection pain in Group P was much higher than that in Group R (80.5% vs 2.4%, p < 0.001). The incidence of other adverse events, such as low SpO_2_, bradycardia, and hypotension in Group R was lower than that in Group P (*p* < 0.05). During the examination, 15 and 20 patients in Group R and Group P, respectively, exhibited slight body movement such as visible bending of the hand or movement of the head, but this did not interfere with the operation or cause dropout from the research (*p* > 0.05).

### Changes in circulation

**Fig. 2:**
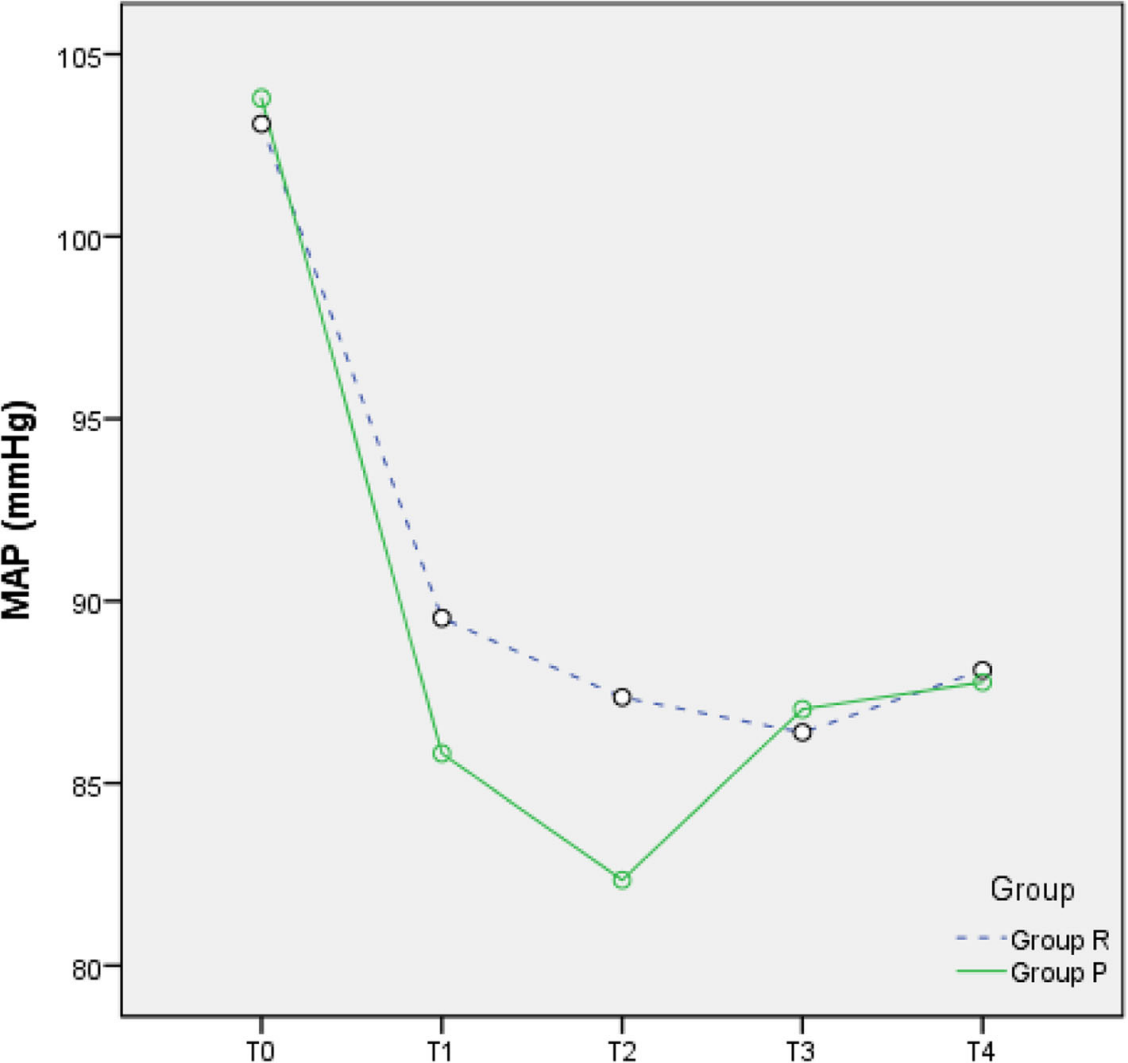
Mean arterial pressure (MAP)-time graph

**Fig. 3:**
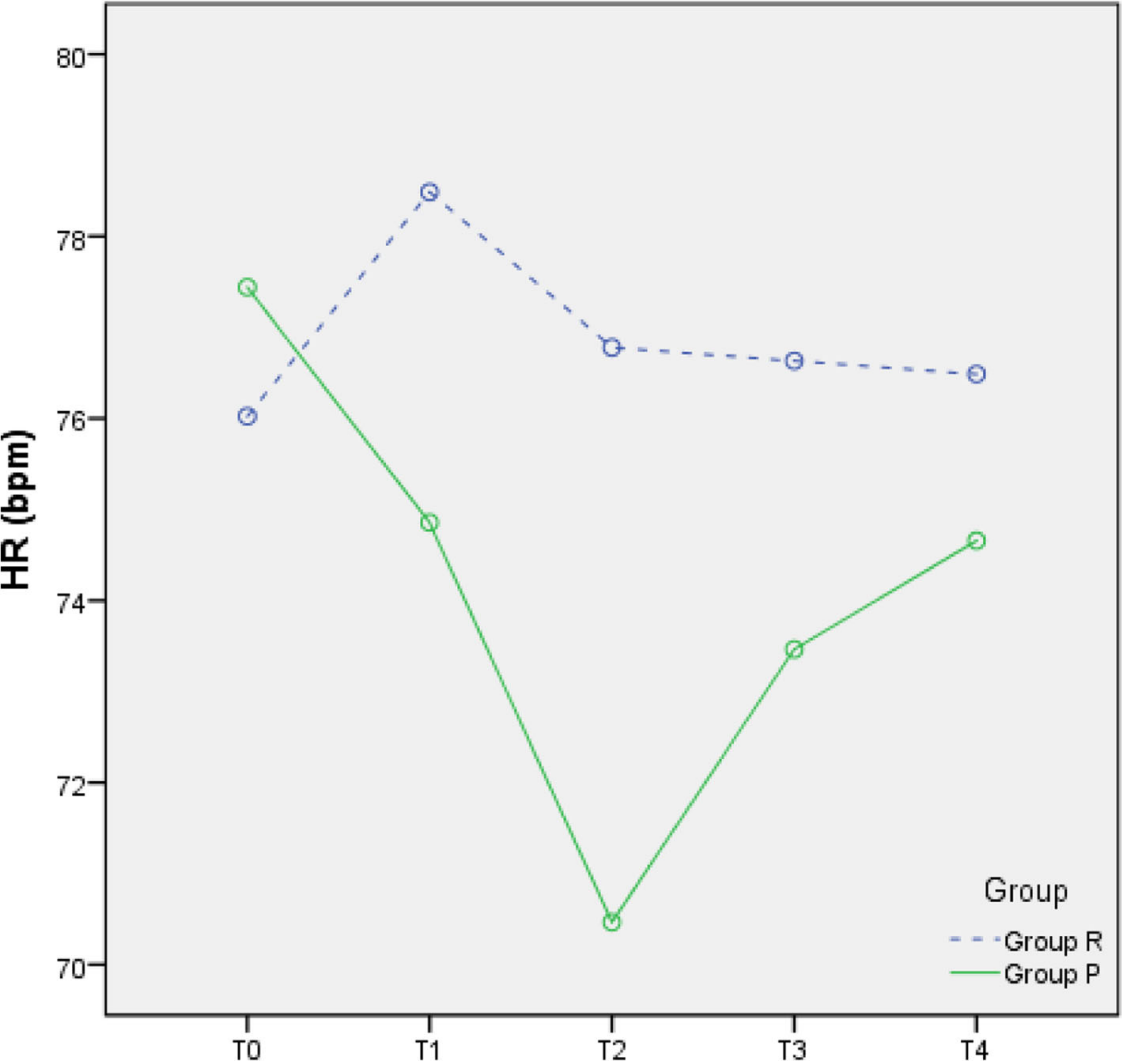
Heart rate (HR)-time graph

**Fig. 4:**
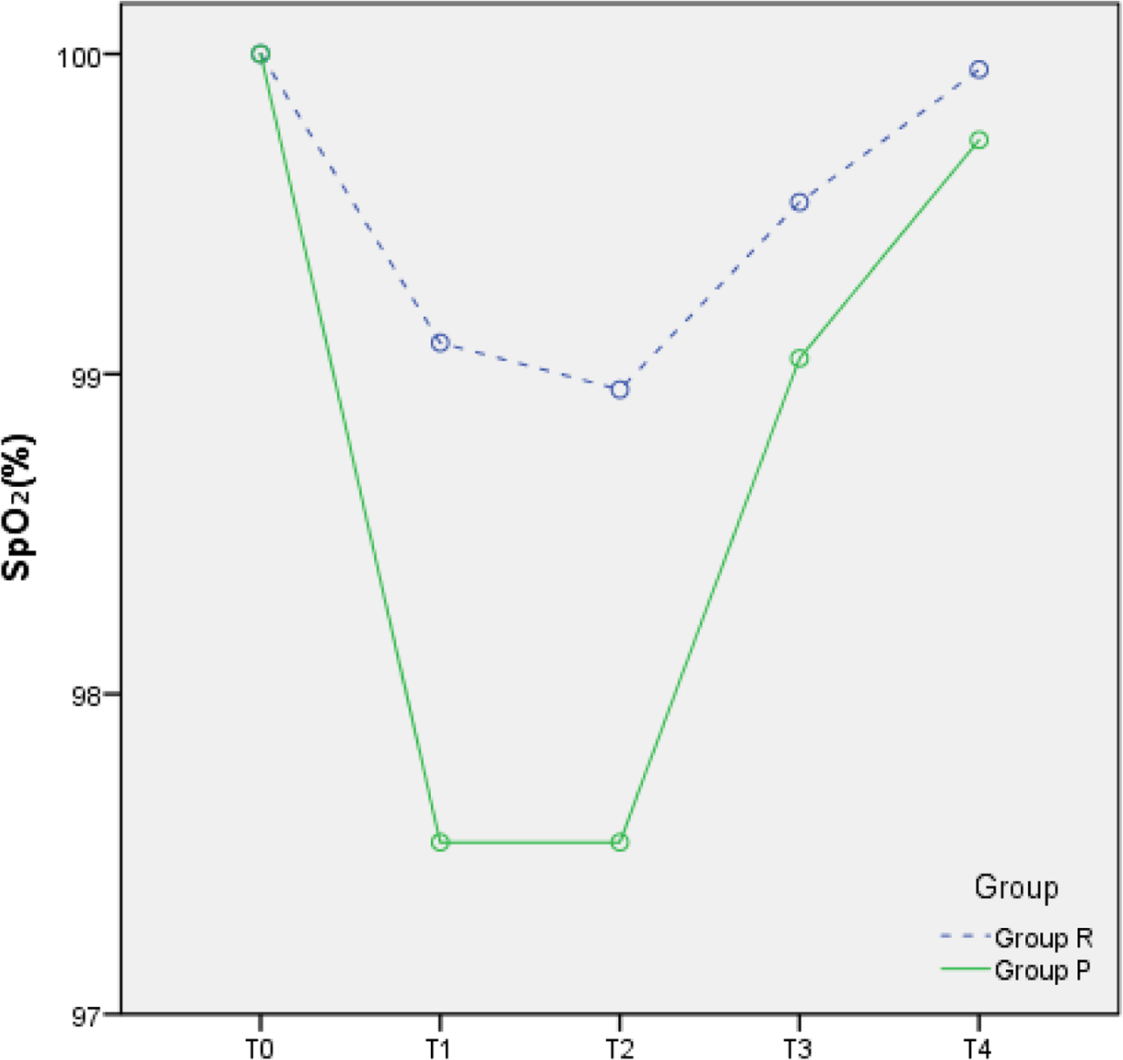
SpO_2_-time graph

Compared with T0, the MAP, HR, and SpO_2_ at T1–4 were all reduced in the two groups (all *p* < 0.05), but all values were within the clinically normal range (Figs. 2, 3, and 4). During the anaesthesia, only one patient in Group P had bradycardia (HR < 60 bpm), but this condition improved rapidly. Compared to Group P, Group R showed less fluctuation in the MAP, HR, and SpO_2_.

### MOAA/S score

**Fig. 5:**
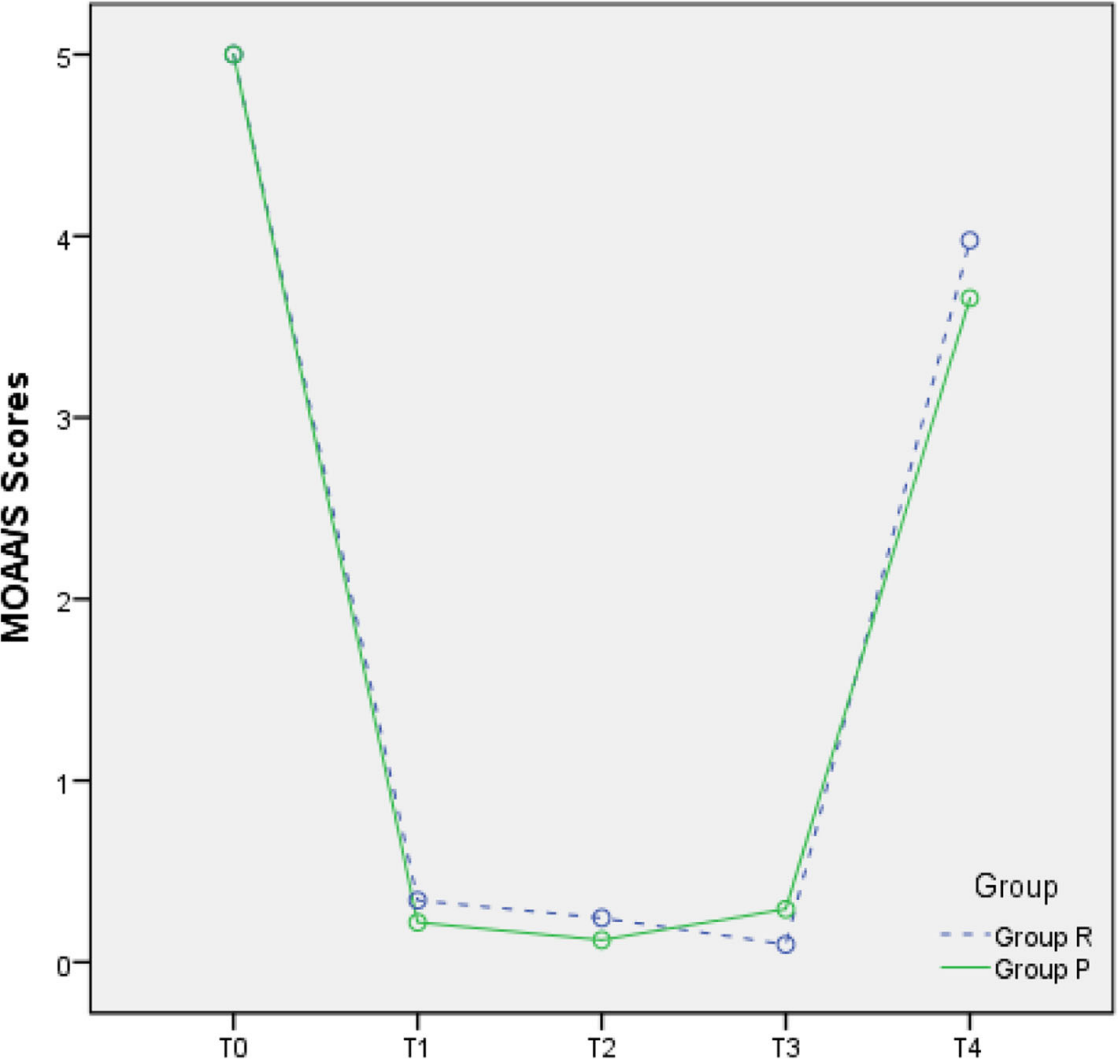
MOAA/S-time graph

In this study, the rate of anaesthesia success in the two groups was 100%, and the patients in the two groups did not require any other medications or withdraw from the study due to insufficient anaesthetic depth. The MOAA/S scores in the two groups during hysteroscopy indicated that the depth of anaesthesia was adequate and effective (Fig. 5).

## Discussion

This trial was conducted to confirm the efficacy and safety of remimazolam besylate versus propofol during hysteroscopy. Based on our data, remimazolam besylate proves to be a safer alternative for anesthesia during hysteroscopy.

Throughout this study, we observed no serious adverse events or adverse reactions in the two groups that would require withdrawal from the trial. The incidence of adverse events in Group R (6/164, 3.7%) was significantly lower than that in Group P (60/164, 36.6%) (*p* < 0.05). Injection pain, postoperative dizziness and low SpO_2_ were the most common adverse events (Table 2, *p* < 0.05).

In a previous multicentre phase III clinical trial in China, 384 eligible patients undergoing colonoscopy were randomly divided into remimazolam and propofol groups. The remimazolam group had a lower incidence of hypotension (46 (23.71%) versus 97 (51.05%)) and respiratory depression (6 (3.09%) versus 32 (16.84%)) than the propofol group [19]. In another prospective, double-blind, randomized, multicentre study that was performed at 30 US sites to estimate the efficacy and safety of remimazolam compared with placebo and open-label midazolam in patients undergoing bronchoscopy, 5.6% of the patients in the remimazolam group had serious adverse events compared with 6.8% in the placebo group [20]. Our experiment further confirmed those results.

Injection pain is one of the most common adverse reactions of propofol in clinical practice. Remidazolam has the same sedative effect as propofol, effectively avoiding the adverse reactions of injection pain to improve patient comfort.

The duration of awakening of patients in Group R (199.0 ± 79.9 s) was longer than that in Group P (59.7 ± 1.2 s). The PACU length of stay of patients in Group R (5.44 ± 1.0 min) was shorter than that in Group P (6.3 ± 1.9 min). Although we observed some statistically differences, these differences did not have a major impact on the clinical procedures. In another multicentre phase III trial, 384 patients scheduled to undergo gastrointestinal endoscopy were randomly assigned to remimazolam and propofol groups. Researchers also found that remimazolam (5.75 min) yielded a faster recovery from sedation than propofol (6.71 min). This is a potential benefit of remimazolam over propofol [21]. It can be speculated that both remimazolam and propofol can meet the required anaesthetic depth and maintenance time for uterine cavity examination, but sedation using remimazolam can avoid the phenomenon of deep sedation observed in the propofol group and has little effect on inhibition of the central nervous system in patients.

Remimazolam has the advantages of rapid onset, a short elimination half-life, and drug metabolism that is independent of liver and kidney function [22]; moreover, it has a specific antagonist, namely, flumazenil [23]. As a benzodiazepine, remimazolam could be evaluated as an anticonvulsant and for use in intensive care sedation. Remimazolam provides effective procedural sedation with superior success rates and recovery profiles compared to midazolam [24]. At present, data comparing remimazolam with propofol are lacking. In previous studies, remimazolam was effectively and safely used in Chinese volunteers [11, 25].

There are some limitations of this study. This was a single-centre investigation, and the sample size was relatively small, which limited the statistical analysis of the two groups of patients.

## Conclusions

Remimazolam besylate proves to be a safer alternative for anesthesia during hysteroscopy. Meanwhile, adverse events caused by propofol, such as low SpO_2_ and injection pain, are largely avoided. This study was a single centre study, and multicentre studies are recommended to reach more relevant conclusions.

## Data Availability

The datasets generated and analysed during the current study are not publicly available due to institutional restrictions but are available from the corresponding author on reasonable request.
