# Correction to: Efficacy and safety of remimazolam besylate versus propofol during hysteroscopy: single-centre randomized controlled trial

**DOI:** 10.1186/s12871-021-01390-x

**Published:** 2021-06-18

**Authors:** Xiaoqiang Zhang, Shuang Li, Jing Liu

**Affiliations:** Department of Anaesthesiology, Mengcheng County No. 1 People’s Hospital, Mengcheng, 233500 Anhui Province P. R. China

**Correction to: BMC Anesthesiol 21, 156 (2021)**

**https://doi.org/10.1186/s12871-021-01373-y**

Following publication of the original article [1], the authors reported an error in the image of Fig. 1 wherein some data are cut off. The correct figure is shown below.

**Fig. 1:**
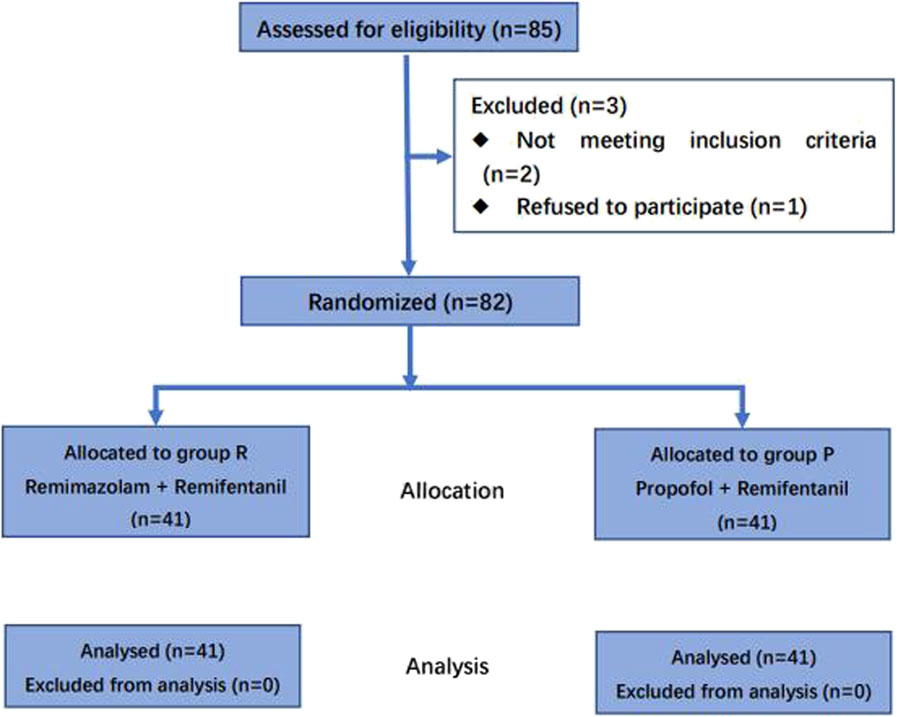
Flow diagram of the study

The original article has been corrected.
